# Analysis of the polyester clothing value chain to identify key intervention points for sustainability

**DOI:** 10.1186/s12302-020-00447-x

**Published:** 2021-01-06

**Authors:** Cristina Palacios-Mateo, Yvonne van der Meer, Gunnar Seide

**Affiliations:** Aachen Maastricht Institute for Biobased Materials (AMIBM), Faculty of Science and Engineering, Maastricht University, Urmonderbaan 22, 6167 RD Geleen, The Netherlands

**Keywords:** PET, Textiles, Value chain, Environmental sustainability, Microfibers, Pollution, Recycling, Life cycle

## Abstract

Clothing is one of the primary human needs, and the demand is met by the global production of thousands of tons of textile fibers, fabrics and garments every day. Polyester clothing manufactured from oil-based polyethylene terephthalate (PET) is the market leader. Conventional PET creates pollution along its entire value chain—during the production, use and end-of-life phases—and also contributes to the unsustainable depletion of resources. The consumption of PET garments thus compromises the quality of land, water and air, destroys ecosystems, and endangers human health. In this article, we discuss the different stages of the value chain for polyester clothing from the perspective of sustainability, describing current environmental challenges such as pollution from textile factory wastewater, and microfibers released from clothing during the laundry cycle. We also consider potential solutions such as enhanced reuse and recycling. Finally, we propose a series of recommendations that should be applied to polyester clothing at all stages along the value chain, offering the potential for meaningful and effective change to improve the environmental sustainability of polyester textiles on a global scale.

## Introduction

The global volume of fiber production for textile manufacturing reached 110 million metric tons in 2018 [1] making clothing and textiles the fourth largest industry in the world [2]. About two-thirds of all textile fibers are synthetic, and more than half are made from oil-based polyester [1]. Fiber production for textile manufacturing has doubled in the past 20 years even though the population has only grown by 25% over the same period [3]. This increase, which poses severe challenges to sustainability, can be correlated with fast fashion trends in which consumers expect new products in stores almost every week, while more than 30% of the clothes purchased in Europe have not been worn for at least one year [4]. At the same time, the longevity of clothing has declined, with 2019 estimates in Germany suggesting an average lifetime of only 4.4 years [5].

The combination of increased consumption and shorter garment longevity has led to an increase in global textile waste, which rose to ~ 92 million tons in 2015 [6]. The textile industry also generated 1.7 billion tons of CO_2_ emissions in 2015 and consumed 79 billion cubic meters of water, which is detrimental to the environment and causes pollution that may put human health at risk. Furthermore, factory workers in the textile industry have a higher than average prevalence of respiratory diseases and allergies [7]. In a “business-as-usual” scenario, the quantity of textile waste and corresponding resource consumption and emissions will increase 50% by 2030 [6]. In order to prevent this and improve sustainability, a comprehensive analysis of the textiles value chain is required to identify key points for intervention.

The overall value chain for all fiber materials has been reviewed [8, 9]. However, given the large share of polyester textiles, it is necessary to understand the sustainability of this material in particular and set targets for improvement along this specific value chain. In this article, we therefore discuss the life cycle of conventional polyester and the unsustainable factors at each stage of the value chain as a starting point to define the measures needed to achieve improved environmental sustainability. First, we explain the production of a polyester garment, from raw material extraction (mainly crude oil) to textile confection and distribution. We then consider the use phase, including state-of-the-art information concerning issues such as microfiber release. We also describe different disposal routes and the latest recycling technologies. Recommendations to achieve improved sustainability along the value chain are presented throughout the text and are summarized at the end in the form of three tables.

The sustainability of value chains can be assessed according to the three dimensions of the triple-bottom line: economic, environmental and social impact [10]. Here we focus on the environmental impacts of the current polyester apparel value chain, including manufacture, use and waste management. Environmental impacts include greenhouse gas emissions (also described as the carbon footprint or climate change impact), other emissions to air, emissions to water and land, depletion of resources, non-renewable energy use, land use, water use, and reduced ecosystem quality. Social and economic sustainability are not discussed in detail, although some aspects linked to environmental impacts are mentioned, such as the effect of toxic emissions on health. Polyethylene terephthalate (PET) is currently the predominant polyester material [11]. Accordingly, when we refer to polyester fibers, textiles and garments, this means PET unless otherwise stated.

## Production phase

**Fig. 1:**
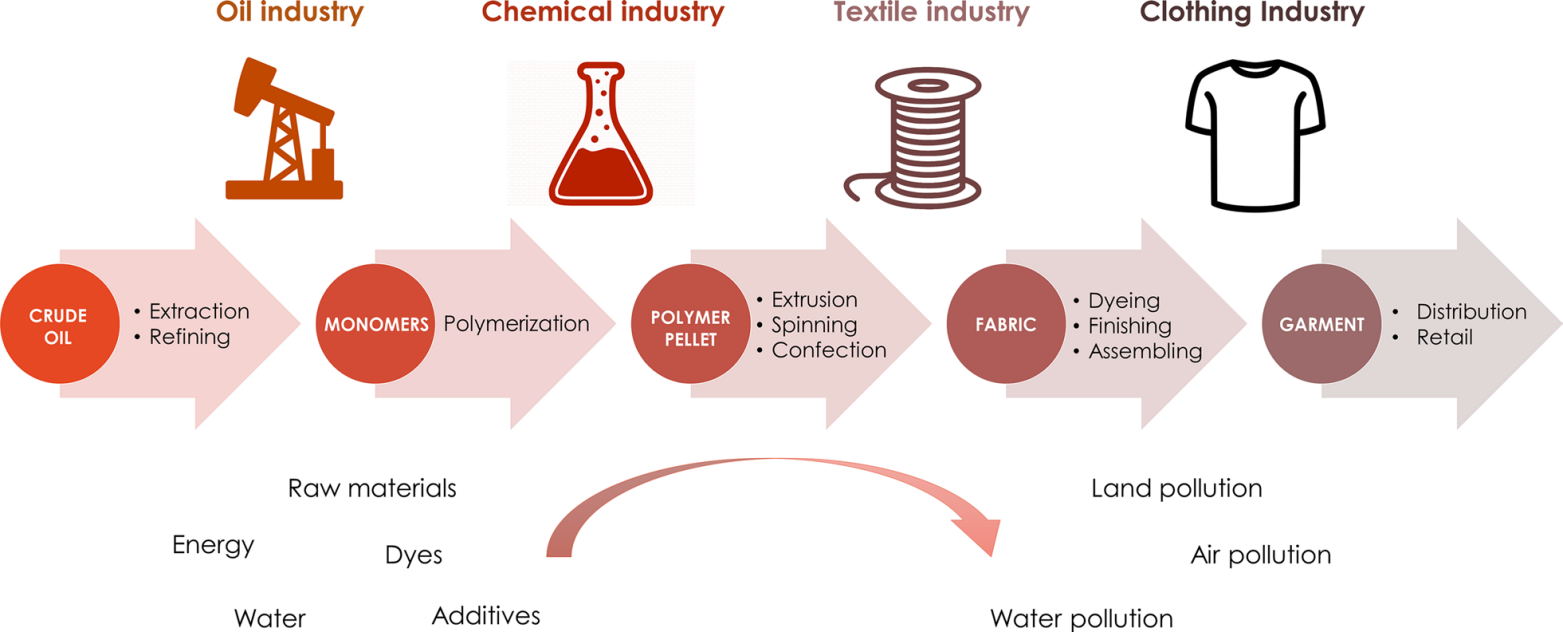
Conventional value chain for polyester garments

The different industries involved in the conventional value chain for polyester apparel are summarized in Fig. 1. The value chain begins with the oil industry, which extracts and refines the crude oil to generate building blocks used by the chemical industry to produce PET and other chemicals (additives). The chemical industry then supplies PET pellets or chips to the textile industry, which converts the pellets into fibers by extrusion and spinning, and then into fabrics by knitting or weaving. This process also involves the incorporation of dyes and additives to impart particular qualities to the fibers and fabrics. Finally, the clothing industry cuts and sews the fabric into garments and makes them available in retail stores.

All these steps require significant amounts of energy, as much as 125 MJ/kg polyester fiber [12], which results in the emission of 27.2 kg CO_2_ eq/kg polyester woven fabric [8]. Furthermore, the poor management of residues along the supply chain can cause soil and water pollution via the direct release of wastewater containing dyes and/or chemicals into nearby water bodies. This not only affects the environment but also the health of the communities living nearby.

The dyeing and finishing step is ranked first in terms of environmental unsustainability, considering the following five impact indicators: climate change, freshwater withdrawal (which includes water use and emissions to water), depletion of resources, ecosystem quality, and human health [8, 13]. Yarn preparation is ranked second, followed by fiber production (including raw material extraction and polymerization). The stage of the manufacturing process that less impact seems to have in the environment is distribution. Each of these steps is considered in more detail below.

### Raw material extraction and processing

The production of conventional polyester apparel starts with the extraction of crude oil. This non-renewable fossil fuel resource consists of thousands of different organic compounds, including pure hydrocarbons, and molecules with functional groups containing oxygen, nitrogen, sulfur and certain minerals [14]. This mixture is trapped within rock layers deep underground and is extracted by drilling and pumping, which consumes energy and disrupts the surrounding ecosystem.

Because crude oil is such a complex mix, it must be refined and processed to obtain the building blocks of PET, namely ethylene glycol and terephthalic acid (TPA). This is achieved by heating, distillation and other processes that release harmful toxins such as BTEX compounds (benzene, toluene, ethylbenzene and xylene), particulate matter, nitrogen oxides (NOx), SO_2_ and CO. If not controlled, these compounds can contribute to air pollution and global warming [15].

Furthermore, oil and the chemicals used during extraction are often spilled. For example, 2811 spills were reported by oil and gas companies in Colorado, New Mexico and Wyoming in 2019, nearly eight per day, amounting to 23,600 barrels of oil and 170,223 barrels of wastewater [16]. This has detrimental effects on the surrounding population and the environment. In Nigeria, oil extraction has damaged soil fertility, destroyed wildlife and affected fishing activities due the spillage of toxic compounds [17]. Given that most residents of the Niger Delta depend on agriculture and fisheries, this has severely limited their income and affected their lives. Furthermore, high levels of heavy metals such as chromium, lead and arsenic were found in their food, posing serious threats to health [18]. The better management of oil resources to reduce the number and severity of spills would improve the surrounding environment and thus the livelihood and health of its residents. However, the major constraint is the lack of enforcement of existing regulations [18].

The building blocks for PET can also be obtained from recycled materials (see “Recycling” section) or renewable resources such as CO_2_ and biomass. Given the abundance of CO_2_ and the threat it poses, carbon capture and utilization is now considered not only viable but possibly essential for future value chains. Laboratory-scale electrochemical systems can efficiently convert CO_2_ into chemical building blocks (such as ethylene glycol) to obtain polymers [19] but more research and development is required to optimize and scale up this technology [20]. Whereas CO_2_ conversion technology is not yet mature, ethylene glycol has been produced from biomass for many years, and industrial biobased processes for the production of TPA are emerging [21]. However, the economic feasibility of biobased production is currently limited [22]. As a consequence, less than 1% of PET production in 2018 was partially biobased, meaning that ethylene glycol was derived from biobased sources, but TPA was still produced from oil [23].

It is important to note that renewable materials are often considered sustainable, but this may or may not be the case depending on the raw material, production process and energy source. It is therefore necessary to verify the environmental sustainability of biobased and CO_2_-based solutions using quantitative evaluations, such as life cycle assessment (LCA). For example, a comparison of biobased TPA (produced from corn, sugarcane and orange peel) and TPA produced from oil [24] revealed that first-generation raw materials (corn and sugarcane) had a similar environmental impact to oil, mainly due to the depletion of resources and the extra land required for crop cultivation. In contrast, the biobased route involving second-generation materials, specifically the upcycling of side-streams such as orange peel, achieved the most sustainable solution with the lowest environmental impact because it did not involve resource extraction or land use and made use of resources that would otherwise be wasted.

Key recommendations to improve the sustainability of polyester manufacturing at the raw material stage therefore include phasing out the use of fossil fuels as a material source for PET production and for the provision of energy. The raw materials can be replaced with recycled chemicals and/or renewable feedstocks, depending on which has the smallest environmental footprint (verified through LCA) and the energy requirements can be provided by renewable sources.

### Polymer synthesis

**Fig. 2:**
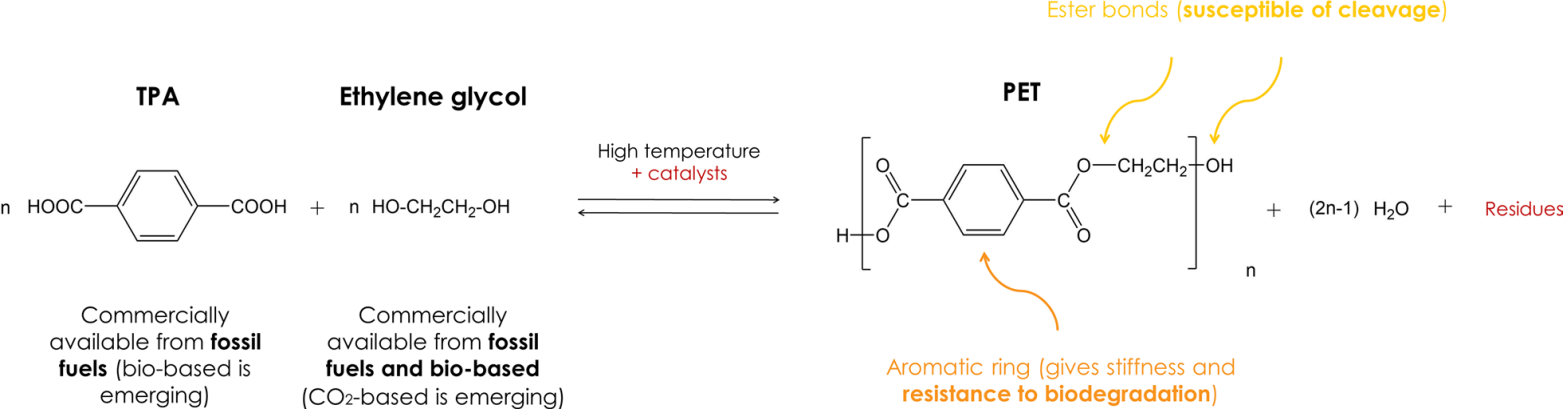
Synthesis of PET from ethylene glycol and TPA

Ethylene glycol and TPA react by condensation to form ethylene terephthalate units, which are then linked via ester bonds (CO–O) to form the long chains of PET (Fig. 2). In theory, ester bonds can be hydrolyzed, which means PET can be de-polymerized, but the large aromatic ring gives PET notable stiffness and strength, especially when the polymer chains are arranged in an orderly manner as in the case of textile fibers, making PET highly resistant to biodegradation at its end-of-life phase [25].

The poly-condensation process requires high temperatures (up to 290 °C) and catalysts such as metal oxides or metal acetates [25]. The wastewater contains chemical residues, and appropriate disposal is therefore necessary. The smart management of resources and residues can help to improve this process, and the use of renewable energy is recommended where possible because the generation of high temperatures results in significant CO_2_ emissions if fossil energy is used. In the final step, PET is compressed into pellets for sale. These pellets are considered a subgroup of microplastics and cause detrimental effects in the environment if spilled during distribution [26].

### Textile production

**Fig. 3:**
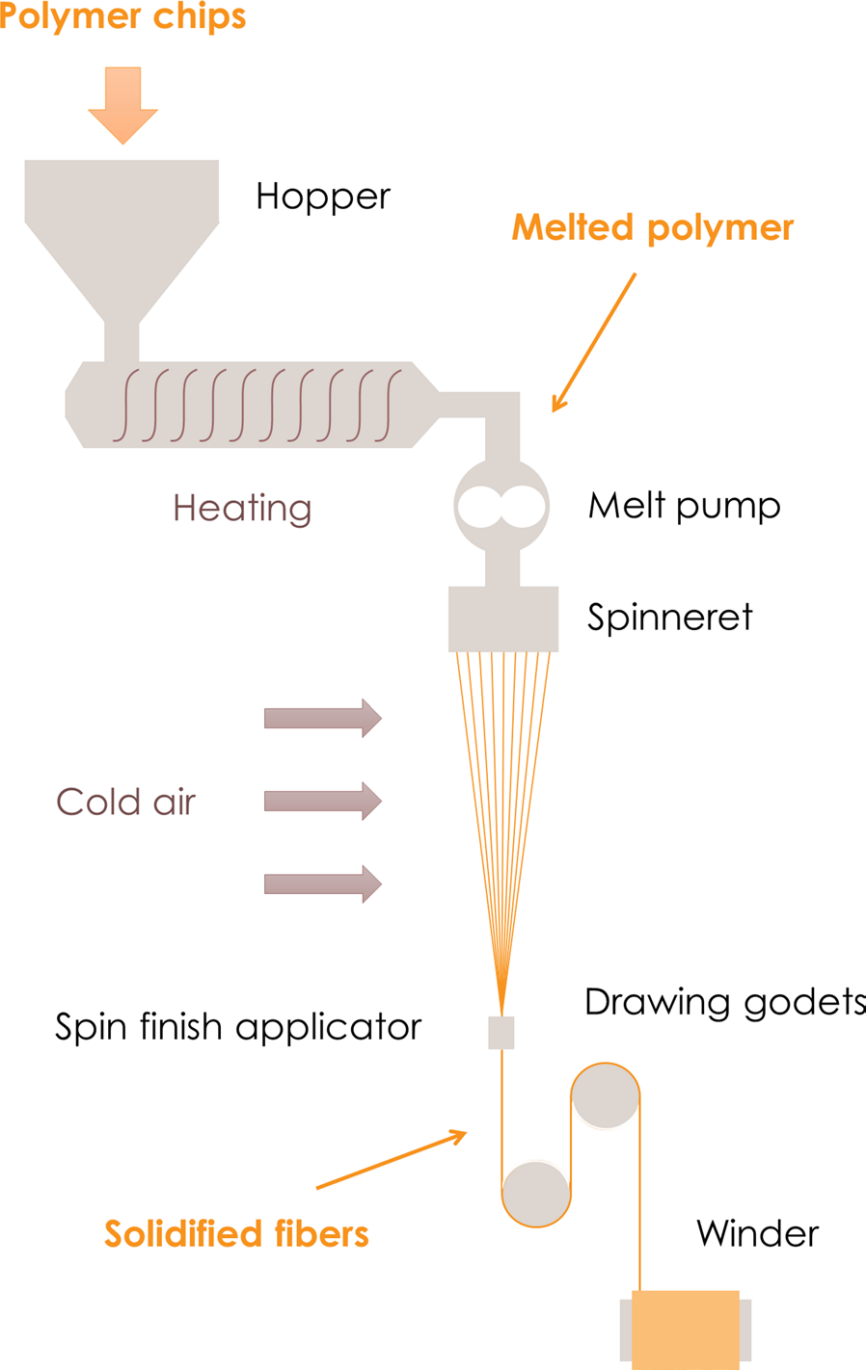
Spinning process from pellet to fiber

PET pellets are melted, extruded and spun into filaments (Fig. 3). These filaments are then subjected to a thermal drawing process to improve mechanical properties such as tenacity. During the drawing process, PET molecules are reoriented in the fiber direction and crystallize. The crystallinity of the fiber therefore depends on the applied draw ratio [27].

Drawn filaments are then combined and further processed in different ways to form yarns with specific characteristics [28]. There are many ways to combine filaments into yarns, depending on the final application of the textile. Yarns can have a high twist (which provides structural integrity), a low twist or no twist. They can be prepared from short *staple* fibers or longer *infinite* filaments. Similarly, yarns can be texturized at different levels to make them softer or more flexible, which can be achieved by the thermal or mechanical deformation of individual filaments. The total amount of energy consumed during this step depends on the thickness of the yarn, because thinner yarn has a lower energy demand per kilogram [29]. Regardless of the yarn properties, renewable energy is recommended to reduce CO_2_ emissions.

Yarns are then knitted or weaved to produce fabric, which is confected into garments. This involves pattern-cutting (mechanical or thermal) and sewing. The smarter the pattern-cutting process, the less waste is generated. Unused fabric cut-outs (along with fiber and yarn residues) are known as production waste, which can represent up to 30% of the fabric involved in confection [30]. A smart design process using software that minimizes the size of cut-out pieces and, if possible, recycles this waste back into the textile chain, is already widely used in the textile industry, and sets a good example of sustainable manufacturing [31].

Microfibers are released into the air during garment manufacturing and can stay there as airborne fibers [32]. The term *microfiber*, as understood by the environmental science community, refers to fibers that are 1 µm to 5 mm in length, with a length to diameter ratio greater than 100 [33]. Given the high aspect ratio and surface area of such fibers, compounds that bind to the surface can accumulate as environmental pollutants [34]. Microfibers are considered a subgroup of microplastics and can detach from textiles throughout their life cycle due to mechanical forces.

Factory workers come into contact with microfibers, synthetic dyes and petrochemicals on a daily basis through inhalation or skin contact, putting their health at risk and increasing the prevalence of respiratory disorders (including asthma and interstitial lung disease) and allergies [7]. Long-term exposure (10–20 years) is also associated with a higher incidence of lung cancer [35]. This is analogous to asbestos, a mineral fiber that is banned in many countries [36] due to its harmful effect on the lungs, leading to a specific type of cancer known as mesothelioma [37].

### Dyeing

Pigments and colorants can be applied to textiles at different production stages. They can be mixed with the melted polymer, or added to fibers, yarns, fabrics or garments using different techniques that vary in their environmental impact [11].

The traditionally popular method is batch-dyeing, which consumes up to 150 L water/kg fabric [6]. Here, textile products (fibers, yarns, fabrics or garments) are submerged in an aqueous solution containing dyes and chemicals such as dispersing agents and carriers. Some of these chemicals may be hazardous [38] and the wastewater must be treated before disposal or reuse. Wastewater treatment is common practice in Europe, but other textile-producing countries pump wastewater directly into water bodies [29] causing environmental pollution through emissions to land and water, and thus direct harm to the ecosystem [39]. Approximately 20% of global water pollution is attributed to the dyeing and finishing of textile products [2]. Furthermore, PET fibers are hydrophobic and highly crystalline, so thermal assistance is required during batch-dyeing so that pigments can penetrate the fiber [11]. This emits 2.31–4.14 kg CO_2_ eq/kg finished textile into the atmosphere [29].

A more recent method for the dyeing of synthetic fabrics or garments uses supercritical CO_2_ as a solvent [40]. Non-polar dyes readily dissolve in supercritical CO_2_, avoiding the use of water or chemicals. Furthermore, this method can use CO_2_ captured from industrial emissions and recycle it in a closed-loop system. However, high pressure is required to generate supercritical CO_2_ (170–270 bar) which increases energy consumption [40]. The energy costs and capital investment needed for supercritical CO_2_ dyeing makes this method unappealing for many companies. Only a few offer this technology, for example DyeCoo in the Netherlands.

Another method is dope dyeing, in which pigments are extruded along with the melted polymer so that the resulting fibers are already colored. This saves water, energy and the further use of chemicals, and the environmental impact is therefore 30–50% lower than that of conventional dyeing [41]. Because the fibers are colored at the beginning of the textile chain, a smart system should be implemented to extrude and spin only the necessary quantity of colored fibers, avoiding extra production waste. It is easier to produce non-colored fibers in bulk and dye them on demand later, so dope dyeing is not widely used in the industry.

Both synthetic and natural pigments are compatible with any of the dyeing processes outlined above. Synthetic dyes are used most widely because they are stable and inexpensive, but they persist in the environment [42], and some trigger allergic reactions [43] or even cause cancer [44]. Attention has therefore switched to natural dyes [45], such as curcumin [46] and alizarin [47], which are biodegradable and in some cases bioactive (e.g., with antimicrobial properties) [48]. However, natural dyes offer a limited range of colors and have a lower thermal stability, causing them to degrade more rapidly. They are also more difficult to produce in bulk, making them more suitable for small-scale production [11]. Nevertheless, genetic engineering and fermentation technologies have recently made it possible to obtain natural pigments on a larger scale thanks to dye-producing microorganisms. Although these dyes are still not widely available, companies such as Colorifix (UK) and Pili (France) are currently optimizing and upscaling production, and the Dutch company Living Colors has recently collaborated with Puma to create a demonstrator collection using such dyes.

### Finishing

More than 15,000 chemicals can be used during the textile manufacturing process, including detergents, flame retardants, stain repellents, softeners and carriers [49]. On average, the production of 1 kg of textiles consumes 0.58 kg of chemicals [9]. The residues of these compounds (which tend not to be biodegradable) may be discharged directly into the environment where they spread, even entering the food chain [50]. Many of these chemicals are hazardous to human health, for example brominated flame retardants are endocrine disruptors and neurotoxins [51]. Therefore, the use of certain additives combined with poor wastewater management affects not only the health of textile workers, but also that of the communities living nearby. These issues have encouraged researchers to seek biobased alternatives that are safe and biodegradable. For example, some lignin-based compounds are effective as flame retardants [52] and biobased carriers have also been described for dyeing [53]. However, the challenge for most biobased chemicals is cost-effective and sustainable production [22], which requires meticulous evaluation by LCA.

The sustainability of textiles at the finishing stage would be improved by avoiding the use of hazardous chemicals, which would satisfy circular design practices [54] by allowing clothing to be recycled without polluting the recycling streams. Sustainability would also be increased by reducing complexity, for example by using fewer chemicals and avoiding fiber blends, which is also beneficial in terms of circularity. Such an approach would require transparency (accurate listing of the chemicals and fibers used in each garment) and traceability throughout the value chain, for example by incorporating aspects of blockchain technology [55].

### Distribution

The different steps in the textile value chain are often carried out in different countries or regions. Not all countries have oil reserves, so oil is extracted in one place and transported to another for refinement and the production of chemicals such as PET. The PET pellets may then be shipped to another place for conversion to fibers and/or yarns, which are in turn sent elsewhere for conversion to fabrics, and then somewhere else for dyeing before the fabric is confected into garments. These are then shipped to multiple sites for distribution to retailers.

The transport of raw materials, fibers/yarns, fabrics and garments, and all the chemicals needed at each stage, adds up to a large carbon footprint that contributes to global warming. The transport sector (in general) consumes approximately one-third of all energy consumed in the EU, more than 900 million tons of CO_2_ equivalents per year [195]. It is difficult to determine how much of this can be attributed to textiles, although calculations are available for specific sectors: for example, shipping textile products from China generates 0.16 kg CO_2_ eq/kg textile [29].

As stated above, spillages of oil, chemicals and PET pellets often occur during transportation. Legal enforcement on a global scale could help to reduce spillages (or force remedial action when spillages occur) but overall the best approach to reduce the environmental impact of transport costs is to build shorter supply chains between the industries involved in textile manufacturing. This would also improve traceability. Additionally, the probability that different countries share a similar legal framework for its manufacturing practices is higher in shorter supply chains, and it is therefore easier to hold them accountable.

### Retail

Retail provides jobs all over the world (9% of total employment in Europe in 2010) and represents one of the main gateways to the labor market for young people [56]. For a long time, retail has operated under a fast fashion business model, causing garment consumption to increase and sustainability to fall [57]. More recently, sustainable fashion has emerged as part of the slow fashion movement [58]. This advocates for better purchase options based on:

- an ethical production process,
- a low environmental impact,
- durability of garments (quality over quantity),
- recyclability of garments (circular principles).

The slow fashion trend has also led to *greenwashing*—false claims of sustainability to improve brand reputation [59]. In order to avoid this, traceability must be enforced by strict legislation to preserve the credibility of eco-labeling, which is easier in shorter supply chains as stated above. Another issue is that customer choice is often driven by price and personal preference, even if the consumer is environmentally conscious [60]. Clothing stores should therefore embrace sustainability and include an educational component to assure customers they are getting value for money when purchasing eco-labeled products.

The most sustainable options for polyester garments are recycled and second-hand clothing. However, the former may be associated with poorer quality and the latter are often sold in lower-profile shops [61]. Incorporating reused or recycled clothes among clothes from virgin materials in a regular store could help to destigmatize and normalize such garments. This would also make the purchase of sustainable clothes easier for the customer. Zara and H&M provide examples of this strategy with their JOIN LIFE and Conscious lines, respectively, partly made with recycled clothing from their take-back schemes. However, the percentage of recycled material is not disclosed, leading the Norwegian Consumer Authority to accuse H&M of greenwashing [59]. Furthermore, most global fashion brands are known for their poor working conditions (both for retail and factory staff) and failure to embrace ethical fashion.

Other business models are emerging, such as systems based on pre-orders to reduce pre-consumer waste, and understanding fashion as a service through rental or subscription-rental. Leasing clothes instead of selling them would increase the lifespan of a garment and ensure appropriate disposal at their end of life. The initiative Fashion for Good published a report that confirmed the financial viability of such circular models for established retailers [62], although further research on environmental sustainability is required because rental models would also increase the frequency of laundry and transport.

## Use phase

LCA in the textile industry has traditionally focused on water and energy consumption during the use phase, due to laundry, drying and ironing [63]. The energy efficiency of these processes has significantly improved over the last few years [29] and attention has shifted towards microfiber release from the garment to the environment [34]. Furthermore, the use of laundry detergents has been linked to freshwater pollution and eutrophication [64].

### Depletion of resources

During the use phase, clothes are washed, often tumble dried, and ironed, which uses water and/or electricity. The total consumption of resources will ultimately depend on user behavior (e.g., frequency and temperature of washing, drying method) which varies by region, climate, age and lifestyle [65]. These diverse factors make it difficult to estimate an average annual consumption [66].

#### Water consumption

As part of the ATLETE II project, six laboratories across Europe measured the performance of 50 different models of washing machine rated *A* for energy efficiency. The load capacity was 6 or 7 kg and the project tested different models from all known manufactures in the European market. Tests were performed at 60 °C full load, 60 °C half load, and 40 °C half load [67]. For the full-load tests, the water consumption was 35–50 L per wash, with an average of 49 L. For the half-load tests, water use was only 21.2% lower than the full load.

Water consumption by washing machines in different regions of the world has been evaluated based on data published up to 2006 [65]. The average water consumption per wash was 60 L in Europe (where horizontal-axis washing machines are dominant) and 144 L in North America (where vertical-axis washers are more common). Based on assumed laundry frequencies, this represents 10,000 L per year for European households and 41,000 L per year in North America. Despite the assumptions and the outdated data, these results are qualitatively valuable because they reflect how water consumption per wash cycle depends on equipment (vertical-axis machines consume more than twice the amount of water as horizontal-axis machines) and how annual water consumption is determined by consumer behavior. For example, Japanese consumers often drain greywater from the shower into the washing machine [65]. Greywater reuse is not universal but it is common practice in countries with scarce water resources such as Israel and Australia [68]. If correctly treated and disinfected, greywater can be reused to flush toilets and wash laundry, although the most common application is garden/agricultural irrigation and industrial uses that do not require clean water. Similarly, the European Parliament has recently approved a law for the safe reuse of treated wastewater in agriculture [69]. Rainwater collection for laundry has also been proposed [70]. Furthermore, because rainwater is softer than tap water in Barcelona (where the study was carried out), the use of rainwater for laundry could also reduce the necessary dose of detergent by 62% with a positive knock-on impact on the environment.

#### Energy consumption

About 90% of the energy consumed by washing machines is used to heat the water, so lower-temperature washes use less energy [71]. Previous studies have assumed that clothes are generally washed at 60 °C and then tumble dried [63, 72]. Together with the higher energy ratings of equipment 20–30 years ago, the use phase was declared more environmentally harmful than the production phase. Accordingly, work focused on improving the efficiency of washing and drying machines. This was encouraged by legislation such as EU Directives 96/60/EC and 2010/30/EU, which classified and labeled equipment from *A* (best) to *G* (worst) based on energy consumption [73]. Most washing machines and tumble dryers currently on the European market are rated *A* [29]. The average energy consumption for a full load washed at 60 °C in an *A* rated machine is 0.78 kWh per wash (ranging from 0.56 to 1.05 kWh) with a 17% drop for a half load at the same temperature [67]. Consumer behavior has also changed in the last 20–30 years, with more people washing clothes at lower temperatures (40 °C rather than 60 °C), which reduces electricity consumption by 23% [67]. Some campaigns, such as “I PREFER 30 °C” (2014–2016) led by the International Association for Soaps, Detergents and Maintenance Products (AISE), have encouraged consumers to wash clothes at 30 °C where possible, which saves even more energy. In response to such campaigns, the proportion of European consumers washing clothes at 30 °C increased from 25.1% in 2014 to 31.5% in 2017, and in Belgium the proportion rose to 44.5% [74].

Tumble dryers consume around five times more energy than washing machines [29], but this can increase to 15 times more for cotton fabrics, which take longer to dry than polyester [75]. Air-drying significantly reduces energy consumption during the use phase of any garment, but this is not possible in all climates. Indeed, tumble dryer use is more common in European countries with colder climates and rarer when the climate is warm [8]. Ironing is projected to consume an average of 1.6 kWh per hour [8], which equates to 22–62 Wh for a piece of fabric measuring 40 × 60 cm [75]. However, polyester garments do not require ironing as frequently as other fabrics.

The environmental impact of the use phase in terms of resource depletion has been proposed to depend on the following hierarchy of user choices: (1) air *vs* tumble drying; (2) temperature of washing; and (3) equipment efficiency [76]. These authors argue that consumers with *A* rated machines may wash clothes more frequently and at warmer temperatures in the mistaken belief that their high-efficiency equipment would compensate for these choices, which in sustainability science is known as the rebound effect [77]. Appropriate communication and consumer education on sustainable choices is therefore essential to minimize energy consumption during this phase, also reducing CO_2_ emissions when energy is provided by fossil fuels.

### Environmental impacts related to detergents

The overall environmental impact of laundry depends on the type and amount of detergent used, both in terms of resources consumed during production and the pollution of water and land during the disposal of wastewater. Among four forms of detergent (liquid, powder, capsules and tablets), the production of tablets was shown to generate the highest greenhouse gas emissions [78]. Similarly, the components of the detergent (e.g., surfactants) play an important role because some may be derived from petrochemical sources and others may be biobased alternatives from plants [79].

Once a laundry cycle is finished, detergents remaining in the wastewater are either discharged directly into the environment or partially removed in a treatment plant (to mandated levels) depending on the region. However, given the large volume of laundry wastewater that must be treated, significant amounts of detergent still end up in the environment even after processing, putting aquatic and terrestrial ecosystems at risk [64]. Surfactants and their byproducts reduce water quality and oxygenation, which can severely damage aquatic animals and plants. Furthermore, some detergent components appear to be endocrine disruptors, affecting the reproductive system of fish [80]. Detergents containing phosphates cause freshwater eutrophication, and such products have been banned in some countries [81]. Biobased detergents may be less toxic than their synthetic counterparts [79]. However, further research is needed to determine which types of detergent are more sustainable, taking into account the production stage, the environmental effects of released wastewater, and also the effect of different detergent packaging materials. Sustainable detergents should be effective and affordable to compete with their non-sustainable counterparts.

### Release of microfibers

**Fig. 4:**
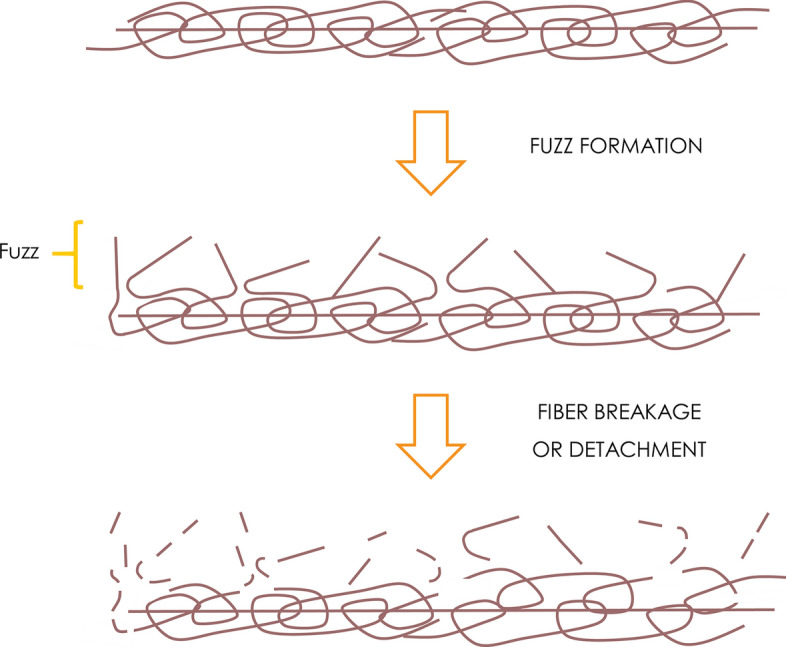
Schematic representation of the proposed source of microfibers. Adapted from [83]

Garments are exposed to various mechanical forces during their use phase. For example, rubbing causes the ends of some fibers to be drawn from the body of the fabric onto the surface, where they appear as fuzz [82]. All textiles produce fuzz to some extent, but the amount produced and the strength of the protruding fibers depend on the properties of the textile, such as fiber material, yarn characteristics, fabric construction and age. If further mechanical or chemical stress is applied into the textile, the protruding fibers might break, leading to the release of microfibers into the environment [83]. Another hypothesis is that the fibers protruding from the surface are simply pulled or loosened from the yarn, shedding without breaking [84]. Regardless of the mechanism (Fig. 4), fabrics that generate more fuzz (more loose ends per unit area) shed more microfibers [85].

Microfibers can be released into the air when garments are worn, and also into the water during washing and drying, in the latter case often accumulating as lint. Approximately equal quantities of microfibers are released during garment wearing and during washing [32]. However, research on the source of microfibers released into the environment has typically focused on detachment during laundry cycles, including the effects of temperature, detergent and the type of washing machine.

Many different factors contribute to fuzz formation and fiber release, so we will assign them to two groups: textile parameters and external parameters (Table 1). In this article, the latter refer solely to the effects of laundry, because the release of microfibers into the air during wearing has not been studied in detail.

**Table 1:** Parameters affecting microfiber (MF) release from clothes during the laundry cycle

Textile parameters	Fiber type	Hydrophilic fibers seem to release more MF than hydrophobic ones Tensile strength might also affect MF breakage	[32, 83, 94]
	Yarn characteristics	Yarns with a higher twist and longer filaments seems to shred fewer MF	[84, 85, 88]
	Fabric structure	Thermally cut fabrics shred fewer MF than mechanically cut fabrics Influence of knitted or woven construction is unclear	[84, 88, 92]
	Age of fabric	Influence is unclear because garments did not undergo realistic aging	[83–86, 89–91]
External parameters	Type of washing machine	Vertical axis seems to contribute to higher MF release, although it may be related to the volume of water used	[65, 91]
	Water volume	Higher water-to-garment ratio seems to increase MF release	[32, 84, 90]
	Speed	No apparent effect on MF release	[90]
	Total duration	No apparent effect on MF release	[89]
	Temperature	No apparent effect on MF release	[83, 86, 88–90]
	Drying	MF release by tumbler dryer seems to be higher than during washing. Difference in MF release between tumbler drying and air drying is unclear	[83]

It is difficult to reach a consensus on the quantity of microfibers shed by different garments during laundry because multiple textile and external parameters act in concert, and there is no standardized method to test, measure or analyze microfiber release, leading to diverse results. For example, one study reported the shedding of 124–308 mg microfibers per kg polyester fabric during a laundry cycle, which corresponds to 640,000–1,500,000 individual fibers [84], whereas another reported the shedding of 0.27–0.46 mg/kg, which corresponds to ~ 80,000 individual fibers [86]. The first report quantifying the release of microfibers in washing machines estimated 1900 microfibers per wash per synthetic garment [87].

#### Parameters affecting microfiber release

##### Textile parameters

Several recent studies have considered the influence of textile parameters on microfiber release [32, 83–86, 88–92]. The testing of polyester garments with different yarn characteristics and fabric constructions revealed that yarns with a higher twist released fewer microfibers than those with lower or no twist, regardless of whether the fabric was knitted or woven [84, 88]. This suggests that tighter yarns make it more difficult for individual fibers to slip or protrude from the fabric*.* Furthermore, fiber length can influence how much fuzz is produced in the first place. Fabrics with yarns made of staple fibers shed more microfibers than those made of continuous filaments, because in the latter fewer loose ends protrude from the surface [85]. The cutting/sewing method used during textile production also affects microfiber release: scissor-cut textiles shed > 30 times more microfibers than laser-cut textiles, because the latter thermally seals the edges of the fabric and thus reduces the likelihood of protruding fibers [92].

Polyester is often blended with cotton in the textiles industry and two studies have considered polyester and cotton garments with the same yarn and fabric construction, both finding that cotton released more microfibers than polyester [83, 84]. This was attributed to polyester having a greater resistance to breaking [83] and to the cellulose fibers of cotton being less hydrophobic [32]. The latter would cause cotton fibers to swell more in water, not only exposing them to breakage but also generating more space for microfiber movement. Another study found that polyester fabrics released more microfibers than cotton, but did not account for different yarn characteristics [86].

Finally, the age of the garment has also been evaluated as a factor influencing the release of microfibers during laundry. The quantity of microfibers released during laundry decreases after the first wash until it reaches a plateau [83–86, 90]. For example, in a study in which polyester garments were evaluated over 10 washing cycles, the first cycle yielded 125 mg/kg of microfibers but this eventually declined to a constant value of ~ 20 mg/kg [84]. A similar constant value of ~ 25 mg/kg was reported in another study [89]. However, these sequential wash cycles did not accurately represent the aging of garments because there was no interstitial use, and therefore little opportunity to generate fuzz. Accordingly, the mechanical aging of polyester garments for 24 h between wash cycles resulted in a 25% increase in microfiber release [91]. Even so, it is not clear whether mechanical aging is an accurate simulation of natural aging, and further testing is required under more realistic aging conditions to determine how microfiber release varies during the life of a garment.

##### External parameters

The effect of different external parameters on the release of polyester microfibers has been tested in both laboratory simulated washers [83, 85, 89, 90] and in real commercial household machines [32, 83, 86, 90, 91]. Home laundering experiments are often used to quantify microfiber release because they offer a realistic scenario, but there is a good correlation between the two kinds of experiments suggesting laboratory models are also representative [83, 90]. The advantage of laboratory studies is that external parameters are easier to control and the washers are simpler to operate and allow the better recovery of samples for analysis [83]. Laboratory studies also address the need for standardization [85].

Home laundry experiments have considered the impact of different types of machines. For example, one study compared microfiber release in vertical-axis machines with a central agitator and horizontal-axis machines [91]. Settings for wash volume, temperature and wash cycle duration were similar in both machines. Speed was only stated for the vertical-axis washer with the central agitator, which shed approximately seven times as many microfibers as the horizontal-axis machine. The authors proposed that the central agitator may have caused more intense movement in the water compared to the horizontal drum, causing more damage to the garments.

Based on the hypothesis that mechanical stress from the laundering processes is responsible for the release of microfibers, polyester garments were tested in washing cycles of 1, 2, 4 and 8 h, to confirm that longer washes lead to more shedding [89]. However, the authors found that a similar amount of microfibers was released regardless of the washing time, and thus the total amount of agitation. Similarly, no significant difference was observed between wash cycles lasting 15 and 60 min [90]. This suggests most microfibers are released within the first 15 min of the wash cycle, which would support the idea that the formation of fuzz during normal wear is a fundamental step required for microfiber release, and that only such loose and protruding fibers would be susceptible to shedding. The main external parameter that affects the detachment of fuzz appeared to be the water volume to garment ratio [90]. The authors conducted several experiments in which the same amount of polyester clothing was washed at the same temperature for the same duration, but in different volumes of water and at different speeds. Larger quantities of microfibers were released in wash cycles that used more water, regardless of the speed/agitation*.* Likewise, the microfiber release rate of 124–308 g/kg clothing [84] increased to 128–1054 mg/kg clothing when the experiment was repeated at a higher water-to-fabric ratio [32]. These results suggest that a higher overall hydrodynamic pressure on the textile may enhance the mobility of microfibers that form part of the fuzz. This may also explain why more microfiber shredding was detected in a vertical-axis washing machine [91], which uses more water than a horizontal-axis device [65]. Finally, several studies have tested the effect of temperature on the release of microfibers from polyester garments, but no significant differences were observed within the temperature range 15–80 °C [83, 86, 88–90].

Although the washing cycle generates significant quantities of microfibers, the use of a tumble dryer produces even more [83]. Air-drying is recommended to reduce energy consumption, but there is evidence that even air drying causes the release of microfibers [93].

#### Fate of microfibers released during the use phase

Microfibers released into the air and water bodies have spread everywhere on the planet, from mountains [95] to rivers [96] in Europe [97], America [98] Asia [99] and the Artic [100]. Given their ubiquitous presence in the environment, microfibers have also entered the food chain and have been detected in many organisms [101] including fruit and vegetable crops [102]. Until standards are adopted, the risk of false characterization or inaccurate quantification must be considered when interpreting scientific studies because the prevalence of microfibers can be overestimated or underestimated depending on the detection method [103–105]. Furthermore, most of the studies discussed below are based on the analysis of individual samples, and these should be extrapolated with caution because longitudinal studies have clearly revealed that microfiber concentrations vary over time and space [106]. Microfibers in the environment should therefore be monitored regularly to gain a clearer picture of the extent of the problem over different spatial and temporal scales [107], although this should not delay the introduction of preventative and remedial solutions.

##### Air pollution

When microfibers are released into the air, they may remain airborne either indoors or outdoors. The testing of different samples for a period of one year revealed that microfiber concentrations are significantly higher indoors (1–60 MF/m^3^) than outdoors (0.3–1.5 MF/m^3^) due to dispersion and dilution [93]. These figures are commensurate with the extent of microfiber shedding from clothes [32] and the fact that we spend most of our time indoors, a situation currently exacerbated by COVID-19. Other household textiles, such as curtains and furniture coverings, also contribute to the production of airborne fibers.

Depending on their size, indoor airborne microfibers may eventually fall to the floor or other surfaces as dust [93, 108]. Airborne microfibers can also fall onto food, which could result in the ingestion of 13,731–68,415 fibers per person per year assuming a cooking and consumption time of 40 min [109].

Outdoor microfibers can be carried by the wind and can fall as dust in the city [110] or in remote areas, as reported for lakes in Mongolia [111] and the Pyrenees [95]. A recent study reported the presence of polyester microfibers on Mount Everest, probably from clothing and equipment based on the detection of greater concentrations of microfibers near major camping sites [112]. Furthermore, precipitation can trap airborne microfibers and deposit them on the ground [95, 113].

Most airborne microfibers both indoors and outdoors were found to be 50–250 µm in length [93], although the method used in this study did not detect smaller fibers, which may also be abundant. Smaller microfibers are more likely to be inhaled, although fibers up to 250 µm in length were also detected in human pulmonary tissues [114].

##### Water pollution

Microfibers have been found in rivers, canals, lakes, seas and oceans [96–99, 115, 116]. They have also been detected in Artic ice [117] and most recently in an Artic freshwater lake [100]. Furthermore, microfibers have been isolated from tap water in a study that tested more than 150 samples from all over the world, with an average concentration of 4.34 particles/L and a maximum of 54 particles/L [118].

Most common textile fibers are denser than seawater: for example, polyester has a density of 1.39 g/cm^3^ [119]. Consequently, microfibers and other microplastics eventually sink (vertical deposition) and have therefore been detected in sediments [115] and in the deep sea [120]. Seafloor currents segregate microfibers (horizontal distribution) and carry them to localized spots of high biodiversity [121]. As they settle (either vertically or horizontally), particles and fibers may be ingested by animals, including those used by humans as food. For example, microfibers have been found in mussels from the Belgian and Dutch coasts [115, 122]. The reported concentration of microfibers in soft tissues varies, reflecting different methods of extraction and analysis. Standardization is required to ensure that studies on microfibers are comparable.

Microfibers have also been detected in a wide range of fish, including 20.5% of Icelandic cod [123], 17.5% of red mullet from the Mediterranean and hake from the Atlantic coast in Spain [124] and ~ 15% of sardine [125]. The same pollutants have also been found in fish-eating birds, such as Mediterranean seagulls [126]. The trophic transfer of microplastics has also been reported from mussels to crabs [127], and from fish to seals [128]. Microfibers were also found in all 102 turtles sampled from the Mediterranean Sea, Atlantic Ocean and Pacific Ocean [129]. The presence of microfibers in marine organisms can pose several problems both individually and for the ecosystem [101, 130, 131]. For example, synthetic fibers ingested by the planktonic crustacean *Daphnia magna* caused an increase in mortality [132]. The fibers were also genotoxic and affected swimming and reproductive behavior [133–135]. This is concerning because organisms at low trophic levels are critical in food chains [136, 137].

Initial research suggested that microfiber pollution in water was mainly caused by laundry effluent, either direct discharges or from wastewater treatment plants (WWTPs) [87, 138]. Laundry effluent was proposed to account for 35% of all global microplastic contamination in the oceans [139]. However, the study did not involve field work and did not account for the deposition of airborne microfibers, thus probably underestimating the problem [32]. Even so, wastewater effluent is still an important source of microfibers in the environment. The analysis of microfibers and other microplastics in seven Dutch WWTPs during 2012 and 2013 revealed that microfibers were the most abundant microplastic in the influent wastewater, confirming the prominent role of laundry [115]. The mean retention efficiency was 72%, which represents the difference in microfiber concentration between the influent and effluent, and estimates the quantity of microfibers retained in the sewage sludge. This suggests that ~ 52 particles per liter are discharged into nearby rivers through the effluent. However, there was significant variation in performance between the WWTPs, probably reflecting differences in treatment methods. A similar study of three Swedish WWTPs using mechanical, chemical and biological treatments revealed a retention efficiency of 99.7% for synthetic microfibers longer than 300 µm, but this fell to 80% for microfibers in the size range 20–300 µm [94].

Differences in retention efficiency between larger and smaller microfibers are relevant because a large proportion of the microfibers released during laundry are less than 300 µm in length [83, 84]. Consequently, 93.3% of the microplastic particles in WWTP effluent were found to be smaller than 300 μm [140]. The analysis of microfibers from WWTP effluent by Raman microspectroscopy revealed that those in the size range 1–10 μm were the most abundant [141]. Technological innovations to increase the retention of smaller microfibers would be desirable [142, 143]. Nevertheless, the presence of even small quantities of microfibers in WWTP effluent means a significant amount is discharged into rivers, due to the large volumes of effluent discharged every day [142]. Assuming a retention efficiency of 98.4%, a WWTP receiving influent from a population of 100,000 would discharge ~ 1 kg of microfibers into the environment every day [91].

##### Land pollution

Microfibers that are successfully removed from wastewater are retained in the sewage sludge. In the Netherlands, sewage sludge is incinerated for energy recovery [115], but in many other countries it is used as a fertilizer because it provides a valuable source of nutrients [144]. This means that 63,000–430,000 tons of microplastics are added to European farmlands every year via sludge applications [144], and microfibers have been detected in farmlands all over the world [145, 146]. Microplastics have even been found in agricultural soils in Germany that have never received applications of sludge, suggesting that significant contamination can be achieved by the use of polluted irrigation water or the natural deposition of airborne microplastic particles [147]. If greywater and blackwater could be treated separately, sewage sludge would no longer contain significant quantities of microfibers and could safely be used in agriculture. Furthermore, greywater treatment could be improved to retain a higher proportion of microfibers. However, this would require significant investment in infrastructure to separate greywater and blackwater at source, and can be regarded as a long-term goal [68].

In addition to the impact on agriculture, the pollution of land with microfibers and other microplastics can affect soil properties as well as the organisms that live in the soil [148–152]. The addition of polyester fibers and other microplastics to soil up to a concentration 2% for 5 weeks or 0.2% for 3.5 months revealed that different microplastics produced different responses in terms of soil properties and soil-dwelling organisms [153, 154]. Particles similar in shape to natural soil particles had less impact in both studies, whereas polyester fibers triggered the strongest effects on soil structure, water dynamics and the activity of soil microbes. The effect of polyester fibers was also investigated in controlled experiments in soil-filled pots as well as a one-year field trial, yielding different results for each scenario and thus revealing the complexity of natural ecosystems [155]. Terrestrial snails ingested less food following the consumption of PET fibers, which was associated with damage to the gastrointestinal walls and lower antioxidant activity [156]. Other microplastics have been shown to pass through the food chain from earthworm casts to chickens, which are in turn consumed by humans [157].

Polyester microfibers also influence plant physiology, triggering the development of longer but thinner spring onion roots with enhanced colonization by soil microbes, and modulating the nitrogen/carbon ratio of the aboveground organs [154]. Similarly, mixing soil with microfibers recovered from a household washing machine inhibited the germination and growth of ryegrass plants [158] suggesting that microfibers in sufficient concentrations could threaten food security and biodiversity [150]. Seeds that germinate in contaminated soil can absorb smaller microplastic particles. In fruit and vegetable crops, microfibers 1.51–2.52 µm in diameter were detected in the edible tissues, with median values of 223,000 particles per fruit sample and 97,800 particles per vegetable, with the highest microfiber burden found in apple and carrot, respectively [102].

#### Effects on human health

**Fig. 5:**
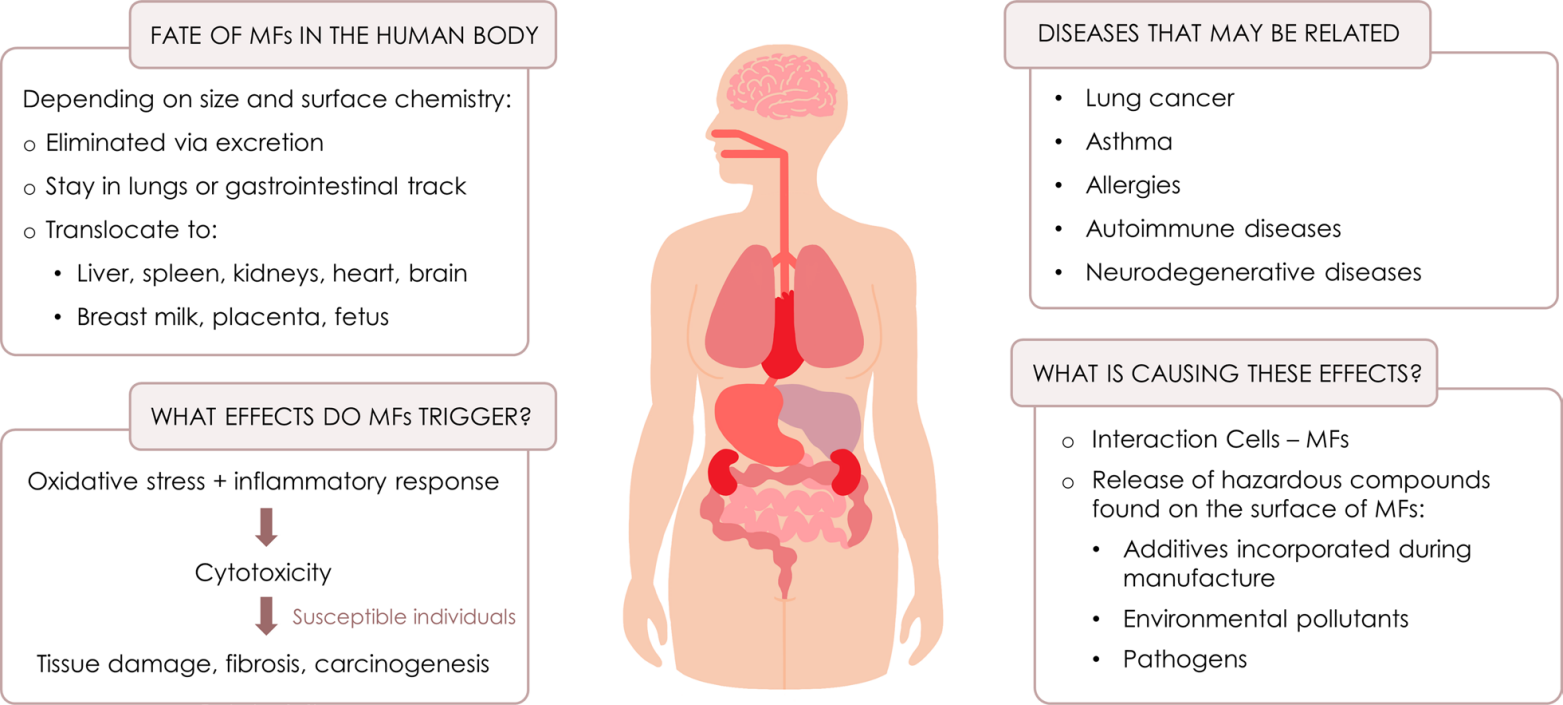
Summary of the effects of microfibers (MFs) on human health

Humans are exposed to microfibers by contact, inhalation and the consumption of contaminated food and drinks. As stated above, there is a clear correlation between microfiber exposure and the health of textile workers, but it is unclear if the remaining population is affected by the generally much lower level of exposure. Multiple tests have been carried out in vitro and ex vivo, as well as in vivo (mostly using mammal models), to measure the uptake of microfibers and determine any toxic effects. Several publications have reviewed the outcomes of these experiments, and more detailed information can be found there [7, 35, 159–162]. The results of these studies are summarized in Fig. 5.

Exposure levels are difficult to measure precisely. The quantity of microfibers ingested depends on the diet and the concentration of microfibers on the surface or within the matrix of ingested food. An intake of 39,000–52,000 microplastic particles per person per year from food and drinks has been estimated in a typical US American diet [163]. As stated above, 13,700–68,400 particles per person per year may also be ingested as microfiber dust settling on food [109]. Based on these numbers, the microfiber intake via the diet is 52,700–73,600 particles per person per year. The number of particles inhaled per year has been estimated in several studies, including relative low ranges of 9500–47,000 particles per person per year [35] but also much higher values of 35,000–69,000 particles per person per year [163]. The gulf between these estimates reflects the different samples and methods used in the corresponding studies, clearly highlighting the need for standardized methods for the evaluation of risk. Further research is also needed to determine the cellular mechanisms of microfiber uptake and toxicity, as well as the impact of different factors such as polymer type, microfiber size and shape, and the presence of additives.

## End-of-life phase

**Fig. 6:**
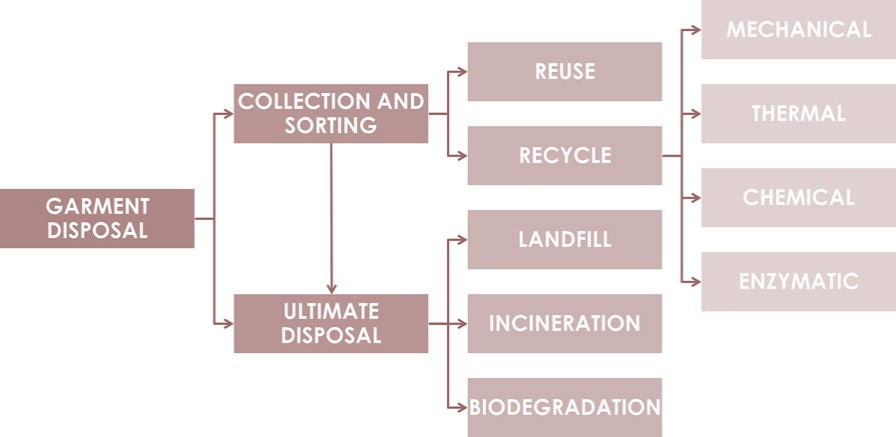
Disposal routes for end-of-life textiles

Depending on how an end-of-life garment is discarded (Fig. 6), the waste material can be eliminated, transformed, or it can accumulate. Most discarded garments are mixed with other household waste, and only a small fraction is properly collected and taken to sorting facilities [54]. There, end-of-life garments are evaluated and sorted depending on reusability and recyclability, and the remainder (as well as garments mixed with household waste) are incinerated or sent to landfill (depending on the legislation), which results in a great loss of resources and potential environmental damage. The proportion of clothing properly collected and sorted was 15–20% in 2017 [9]. However, this figure varies by country. In the Netherlands, of the 305,100 tons of textiles discarded by the consumer in 2018, 44.6% was collected separately in thrift stores or clothing containers and the rest ended up with residual waste, which is incinerated for energy recovery. After sorting, 53% of the correctly discarded clothing was sold for reuse (mostly outside the Netherlands), 33% was recycled, and 14% was incinerated [164]. Therefore, at least two-thirds of discarded clothing is incinerated, mostly due to improper disposal. This reflects both the lack of infrastructure and the lack of knowledge on the correct processes to discard end-of-life textiles. In order to improve recollection rates across Europe, the EU has mandated that all Member States should ensure the separate recollection of textiles in dedicated receptacles by 2025, to enable the better management of discarded clothing [2]. Governments should ensure the proper use of such containers through campaigns and educational programs.

The disposal of clothing in landfills and incineration plants generates pollution, but the more important issue in terms of environmental impact is value loss, because the value of the discarded clothing is replaced by manufacturing new clothes, with a much higher environmental burden than the disposal process alone. If the discarded clothing could be reused or recycled, the production of new garments would be unnecessary because the value of the original materials would be retained. Increasing the reuse and recycling rates for unwanted clothes is therefore a priority for improved sustainability at the end-of-life phase.

Textile waste discarded by the consumer is known as post-consumer waste, but garments are also discarded directly by the retail industry when unsold, returned or defective [30]. This is known as pre-consumer waste, and may account for about one-third of the clothes produced in total [165], although these numbers have not been verified [30]. There is also the production waste mentioned above—the cut-out fabric remnants and yarn residues. Many companies incinerate their unsold garments and cut-offs. For example, the British brand Burberry incinerated $37 million of unsold inventory in 2017 [166]. In order to operate within the European Circularity Plan, some countries such as Spain are starting to draw up legislation that forbids companies from incinerating their pre-consumer waste [167]. France has already approved such legislation, which will come into force from 31 December 2021 [168].

### Reuse

When a garment discarded by a consumer is still wearable or can be made wearable with minor repairs, the practical life of the garment can be extended by transferring it to a new owner via second-hand stores (physical or online) or charities. The reuse of clothes reduces the consumption of resources for the manufacture of new garments as well as avoiding waste. A review of 41 publications on this issue concluded that reuse is the most environmentally beneficial way of disposing of a garment, compared to recycling, incineration and landfilling [61]. However, it is important to ensure that the reuse phase is sufficiently prolonged, to ensure that any impacts from the increased reuse of textiles (such as emissions from the vehicles used for collection and distribution) do not exceed those avoided by producing a lower volume of new textile products [61].

### Landfilling

Polyester garments accumulate in landfills because conventional PET is not biodegradable, resulting in a long post-consumer life even if the use phase is brief. Exposure to the effects of weather over time eventually leads to the fragmentation of fabrics, potentially releasing any harmful additives used during production as well as microfibers. These pollute the land, water and air as discussed above [169]. Landfill was traditionally the major disposal route for textiles, but land scarcity and the threat to human health and the environment has encouraged the selection of other disposal routes. The EU has mandated that a maximum 10% of municipal waste can be consigned to landfill by 2035 [2]. The Netherlands is one of a small number of countries that has already stopped disposing of waste in landfills, and all waste is incinerated or recycled instead.

### Incineration

Incineration is the burning (thermal degradation) of waste, which can be carried out under controlled or uncontrolled conditions, in the presence (combustion) or absence (pyrolysis) of oxygen, and with or without energy recovery [169, 170]. Incinerated waste is not eliminated, but transformed into toxic gases and hazardous residual ash which require further disposal methods. The incineration of PET textiles under different conditions mainly produces CO_2_, CO (probably due to the high oxygen content in the polymer) and benzene, as well as large amounts of TPA, benzoic acid, acetaldehyde, and aliphatic C1–C4 hydrocarbons, and smaller (but still significant) quantities of dioxins and furans [170]. All these compounds are environmental hazards and a threat to human health [171].

Under controlled conditions, some of the toxic emissions can be partly removed, such as the capture of dioxins in active coal filters. A study of 90 incinerator plants in France, with different technologies for air pollution control, revealed there was no specific technique for the abatement of CO or volatile organic compounds such as benzene, resulting in the emission of 43.9 g of CO and 4.68 g of volatiles per ton of incinerated municipal solid waste, 13% of which represents discarded textiles [172].

Nevertheless, incineration is still preferable to landfilling because it does not take up as much space and the impact on air pollution can be partly or fully compensated by recovering energy as heat or electricity, which otherwise would have been produced from fossil fuels [173]. For example, 100% polyester garments can generate 21.2 MJ/kg of recovered energy [174]. Incinerator plants with energy recovery therefore cover 84–90% of their own electricity requirements [172]. However, not every incinerator plant recovers energy from waste [172] and the incinerator infrastructure should be modernized to reduce its environmental impact.

### Recycling

The recycling of synthetic textiles is a broad concept, but all recycling involves a degree of deconstruction. This results in a secondary source of material to manufacture a similar or dissimilar product, thus avoiding the need for virgin raw material. Textile recycling ranges from the recovery of fabric or fibers to the degradation of the material to recover polymers or even monomers, avoiding the need for incineration or landfilling [175]. Despite these benefits, recycled material remains unpopular and virgin material is usually preferred. The market share for recycled polyester was only ~ 13% in 2018 [23]. Part of the reason is the lack of infrastructure and efficient recycling technology in many regions [9]. One challenging step is the sorting of the recollected material. Recollected clothes are a heterogeneous mixture of natural and synthetic fabrics and also blends, differing in quality. Automated sorting has been achieved by analyzing clothes using infrared sensors [176], but this technology is not widely available and most discarded clothes are sorted by hand [30]. Recycling usually targets monomaterial fabrics, which are easier to process than blends, whereas the latter are usually discarded.

Another factor that hinders recycling is the general lack of information about the manufacturing history of discarded garments, including the content of specific additives and dyes. The risk of residual toxic additives in recycled materials limits market growth [61]. The risk depends on the nature of the recycling process, which can include mechanical, thermal, chemical and potentially enzymatic methods, as described below. The ability to recycle fabric, fibers, polymers and monomers depends on which method or combination of methods is used:

- *Mechanical recycling *allows for fabric and fiber recycling. At a global level, this is the most common recycling process because it does not require expensive equipment or reagents [169]. Fibers can be recovered by shredding and pulling, and their reusability then depends on length and quality. Longer fibers can be used along with virgin material to make carpets and rugs, whereas shorter fibers are usually downcycled into insulating or filling materials. Clothing discarded after extensive use and many wash cycles may predominantly contain short fibers of reduced quality. Mechanically recovered fibers are therefore mainly used for downcycling. Whatever the final use, additives may be carried over to the new product [30].
- *Thermal recycling *is often used for polyester and other thermoplastics [30]. The garments are cut and granulated into PET pellets by applying heat (above 260 °C) and mechanical agitation. The polymer pellets can then be used for spinning and extrusion like virgin pellets, and may be used to make new garments or alternative products [61]. Several companies offer thermal recycling equipment, and this recycling method is particularly popular in Europe. However, the shortening of the polymer chains during thermal recycling leads to a loss of quality [177]. Other disadvantages of thermal recycling are the high energy consumption [176] and the carryover of additives to the new product [9].
- *Chemical recycling* is used to recover oligomers or monomers by the depolymerization of PET. The reaction that converts ethylene glycol and TPA into PET is in equilibrium and can therefore be reversed (Fig. 2). This monomer recovery is achieved by hydrolysis [178]. Other methods for oligomer recovery include methanolysis [179] and glycolysis [180]. In each case, the textile is first cut into pieces and then submerged in a chemical solution for high-temperature depolymerization with specific catalysts [181]. Additives and dyes dissolved in the solution must be removed and disposed of properly, and then the monomers or oligomers can be purified and repolymerized, yielding PET pellets and fibers of the same quality as virgin polyester. Recent work to improve the process has focused on the feasibility of chemical recycling at lower temperatures to reduce energy consumption [176]. For example, the Dutch company Ioniqa has developed a glycolysis process that works at temperatures below 200 °C and uses catalysts that can be magnetically recovered and reused, which significantly reduces the costs [182]. Similarly, the Swiss company Gr3n has reduced the costs of hydrolysis by developing a microwave-assisted process that takes on 10 min.
- *Enzymatic bio-recycling *is an emerging technology that uses enzymes to hydrolyze the ester bonds in PET (Fig. 2). Although such enzymes have been studied for many years [183], the natural enzymes are inefficient and protein engineering was required to achieve high monomer yields, as reported by the French company Carbios [184]. Although enzymatic bio-recycling takes a long time per cycle (currently ~ 10 h), its advantages include the low reaction temperature (below 100 °C), the potential reuse of enzymes by immobilization, and the selectivity of the enzyme, which allows the recycling of PET even within blends. Much of the research in enzyme-catalyzed PET depolymerization has focused on PET from bottles or packaging rather than fibers, the latter being more challenging due to the higher content of hydrolysis-resistant crystalline regions [185]. Mechanical, thermal or chemical pretreatment methods may therefore be necessary for the complete enzymatic depolymerization of PET textile waste [177]. Such processes should be evaluated by LCA to ensure that the environmental impact of pretreatment does not offset the environmental benefits of enzymatic recycling.

Deconstruction methods to recover polymers, oligomers or monomers are recent innovations that have yet to be applied to fabrics on a large scale. Most recycled garments are therefore downcycled into products such as insulating and filling materials, and only 1% of textile waste is currently recycled into new clothing [9]. This is not necessarily undesirable, because fabric and fiber recycling still avoids the use of virgin PET to manufacture insulating and filling materials [61]. Ultimately, a series of changes is needed to increase recycling rates, including (1) a more efficient collection strategy that promotes compliance; (2) more efficient sorting; and (3) the introduction of circular design principles (replace hazardous chemicals, reduce product complexity, and improve process transparency) to ensure that recovered materials can be identified and handled in a safe and appropriate manner.

### Controlled biodegradation/biotransformation

Although PET fibers are not regarded as naturally biodegradable, the application of enzymatic cascades [186] or microorganisms [187] has the potential to accomplish this process. In a controlled bioreactor, the first step would be similar to enzymatic bio-recycling, yielding oligomers and the monomers TPA and ethylene glycol. Subsequently, further decomposition could achieve biodegradation to final products such as CO_2_, methane and water. Microorganisms in the bioreactor could directly use the monomers or the CO_2_ as a source of carbon to increase their biomass [188] or to produce value-added compounds [189] For example, a strain of *Pseudomonas putida* has been engineered to efficiently convert ethylene glycol into the biodegradable polymer polyhydroxyalkanoate [190]. Given the large quantities of textile and plastic waste generated every year, the controlled biodegradation or biotransformation of PET may be a promising concept for the future.

## Key points for environmental sustainability

In this article, we have presented a qualitative analysis of the life cycle of polyester clothing, which currently involves the unsustainable depletion of resources and the generation of polluting emissions (among others contributing to climate change). The pollution not only damages the environment, but is also a threat to human health. In order to make clothing more sustainable, we recommend several actions that should be implemented during the production (Table 2), use (Table 3) and end-of-life (Table 4) phases. This requires the involvement of multiple stakeholders: governments and NGOs, industry, researchers, and consumers. The new measures should be encouraged through a mixture of legislation, economic incentives, funding, education and communication, because single measures will not suffice. For example, education alone may not promote universal change among consumers, but it is important that the public understands why specific measures are needed, otherwise there will be a lack of cooperation [138]. There are many examples of appropriate and timely legislative decisions that placed society on the path to sustainability, such as the recovery of the ozone layer following the ban on chlorofluorocarbons [191] and the more recent recovery of marine populations, habitats and ecosystems in some regions following direct interventions [192]. In the context of waste management, further examples include European Directive 2000/76/CE, integrated into European Directive 2010/75/EU, which enforced the technological development of incineration plants (although further progress is needed to modernize the incinerator infrastructure). Furthermore, EU Directives 96/60/EC and 2010/30/EU contributed to sustainable energy by encouraging the manufacture of *A* rated washing machines and other domestic appliances. New directives are now needed to wean society off the consumption of energy from fossil fuels during every phase of the textile value chain. This can be achieved by a mixture of stricter CO_2_ taxes on companies, the discontinuation of fossil fuel subsidies and incentives to encourage switching to renewable energy sources and energy-efficient machinery. Other recommendations to increase the environmental sustainability of textiles include the fitting of in-drum devices or external filters to washing machines to prevent microfiber shedding or to capture the fibers that are released. Six such devices have recently been tested, revealing that the commercial filter XFiltra was able to retain 79% of microfibers in the greywater whereas the in-drum Guppyfriend bag helped to reduce microfiber shredding by 54% [193]. The combination of both devices would have a significant impact on the volume of microfibers released during the laundry cycle.

**Table 2:** Key recommendations to improve the sustainability of polyester garments during the production phase of the value chain. GHG = greenhouse gas

Issue	Goal	Measure	Action
Inputs	Phase out fossil fuels as source of energy	Strict taxes on GHG emissions	
		Discontinue buying/selling quotas on GHG emissions	
		Discontinue subsidies for use of fossil fuels	
		Incentivize use of renewable energy	
	Phase out fossil fuels as source of materials	Prioritize recycled PET pellets for production of new polyester fibers	
		Further investigate the environmental impacts, safety and economic feasibility of: Renewable monomers ethylene glycol and TPA for the production of PET pellets Renewable polyesters such as polylactic acid Renewable dyes and other chemicals	
		Optimize and upscale the use of renewable feedstock	
		Consider materials other than polyesters that may be more sustainable	
	Reduce water consumption	Prioritize dyeing/printing methods that require less water	
		Encourage water recycling	
Outputs	Reduce water pollution	Impose wastewater treatment	
	Reduce microfiber release	Prioritize compact yarn structures (high twist, longer filaments)	
		Prioritize thermal cutting methods	
	Reduce waste	Improve management of chemical residues	
		Smart design through digital tools to reduce cut-out pieces	
		Recycle cut-outs back into the supply chain	
Design	Design for durability and recyclability	Work with high-quality materials for durable clothing	
		Discontinue use of hazardous chemicals and dyes to increase recyclability	
		Avoid blends when possible to increase recyclability	
Transport	Supply chain optimization	Shorten the supply chain	
		Implement traceability through digitalization	
		Reduce oil, chemical and polymer pellet spills	
Retail	Make the purchase of sustainable clothes easier	Implement strict eco-labeling	
		Include reuse and recycled garments in regular stores	
		Place retail stores at accessible locations	
	New business models	System based on pre-orders	
		Fashion as a service (subscription or pay-per-use)	

Legislation  Economic incentive or funding  Education or communication

**Table 3:** Key recommendations to improve the sustainability of polyester garments during the use phase of the value chain

Issue	Goal	Measure	Action
Resources	Phase out fossil fuels as source of energy	Incentivize use of renewable energy in the household	
	Reduce energy consumption	Improve machine efficiency	
		Wash at lower temperatures	
		Avoid tumble drying when possible	
	Reduce freshwater consumption	Switch to horizontal-axis washing machines	
		Improve and encourage greywater recycling systems within households or laundry stores	
Detergents	Produce more sustainable detergents	Determine which type of detergent is more sustainable considering: Production Packaging Effects on the environment upon release Effects on microfiber release	
Microfibers	Prevention of microfiber release from clothing	Wash at full load to avoid high water volume to garment ratios	
		Standardization of quantifying methods to facilitate more informative research	
	Retroactive solutions for microfiber release from clothing	Use filters to capture microfibers in washing machines	
		Develop air filtration systems to remove airborne microfibers indoors	
		Bioremediation: can enzymes or whole microorganisms be added to agricultural soils to degrade PET without disturbing soil properties or causing other side effects?	

Legislation  Economic incentive or funding  Education or communication

**Table 4:** Key recommendations to improve the sustainability of polyester garments during the end-of-life phase of the value chain. GHG = greenhouse gas

Issue	Goal	Measure	Action
Resources	Phase out fossil fuels as source of energy	Strict taxes on GHG emissions	
		Discontinue buying/selling quotas on GHG emissions	
		Discontinue subsidies for use of fossil fuels	
		Incentivize use of renewable energy	
Pre-disposal	Reduce volume of discarded clothes	Promote reuse and mending	
Disposal	Improve collection of textiles	Provide information to the public on the correct processes for textile disposal and recycling	
		Facilitate disposal by increasing the number of collection containers	
		Increase the number of retail stores with take-back schemes	
	Improve sorting facilities	Invest in new sorting technologies	
Recycling	Reduce material rejection	Improve transparency concerning the additives used during manufacturing	
	Improve monomer recycling technologies	Can chemical recycling be carried out at lower temperatures?	
		Can enzymatic recycling overcome high crystallinity in fibers in a sustainable way?	
Incineration	Modernization of incineration plants	Improve air pollution control systems	
		Implant energy recovery (both as heat and electricity) in every installation	
Biodegradation/biotransformation	Reduce volume of PET textile waste	Research feasibility and optimize different routes	

Legislation  Economic incentive or funding  Education or communication

## Conclusion

### The problem

Clothing is one of the primary needs of humans. The demand is met by the global production of thousands of tons of textile fibers, fabrics and garments every day. However, the excessive consumption of textiles is detrimental to health and the environment. For example, the full life cycle of 1 kg of conventional polyester fabric has been estimated to release more than 30 kg of CO_2_ equivalents to the atmosphere, contributing to the greenhouse effect and global warming [8]. Most of the environmental burden of the textiles value chain is generated during the production phase, although consumer behavior in terms of laundry routines, purchasing choices and disposal methods also plays a key role [13].

The production phase is characterized by its dependency on fossil resources as a source of materials and energy, and its use of hazardous chemicals and dyes (many of which are also derived from oil). Additionally, approximately 20% of global water pollution is attributed to the dyeing and finishing of textile products. The dyeing and finishing stage is therefore particularly detrimental for the environment, followed by yarn and fiber manufacture. The design of the garment (including the thickness and twist of the yarn, the materials and chemicals required during manufacturing, and the corresponding methods) determines factors such as longevity, recyclability, and (in part) the propensity to shed microfibers. Manufacturing, wearing and washing polyester apparel is a significant source of the microfibers that now permeate the environment, and further research is needed to understand the factors that promote such microfiber release. As for the end-of-life phase, low rates of recovery, poor sorting of textile waste, and the lack of transparency during manufacturing makes it difficult to identify clothing suitable for recycling. Consequently, most discarded clothing is incinerated or sent to landfill, creating pollution and value loss.

### The solutions

A series of changes is needed to reduce the environmental impact of textiles. The main priority is the phasing out of fossil fuels at every stage of the value chain. In terms of energy consumption, this means switching to renewable sources as soon as possible. In terms of materials, polymers, hazardous chemicals and dyes must be replaced with recycled, biobased or CO_2_-based safe alternatives. PET fibers could also be replaced with more sustainable materials where possible. Furthermore, manufacturing methods that require less water and fewer chemicals should be encouraged through a smart design to improve circularity. This should be paired with mandated transparency in the textile manufacturing industry to ensure that all textile products are labeled to identify the fibers and additives used during production. While researchers focus on improvements to avoid microfiber shedding from clothes and WWTPs improve the efficiency of microfiber recovery, simple methods should be encouraged such as installing filters in washing machines and driers. The microfibers recovered from such filters could be recycled along with discarded textiles, although such a process would need to be scaled up and integrated with the broader textile recycling infrastructure. In the last stage of the value chain, recollection rates could be improved by educating the public and by making collection containers more accessible, both of which would encourage compliance.

### The actors

As shown in Tables 2, 3, 4, new legislation, economic incentives, funding and education are needed to encourage sustainability. Governments and industry both have a major role to play. Governments must be the drivers of change to ensure that companies comply with national and international laws. They should provide standards and facilitate access to different tools and resources for more sustainable production and alternatives that promote circularity. Industry must adapt to these changes, and rethink and redirect their strategies by adopting new business models that favor slow and sustainable rather than fast and wasteful fashion. These strategies should be based on state-of-the-art solutions developed by research scientists (academic and industrial) and funding should be made available by government and industry to bring such solutions to scale. Consumers can play their part by adopting sustainable laundry practices and environmentally conscious purchasing and disposal choices. Informed purchasing can be achieved by implementing eco-label standards and by promoting business models based on second-hand and recycled clothing. However, consumers should already be offered the most sustainable options for new clothes in retail stores, which is the responsibility of government and industry.

### The essential

Slow fashion advocates for (1) production processes that do not exploit natural or human resources to expedite manufacturing; (2) conscious consumption that achieves a longer product lifespan; and (3) the disposal of garments in a manner that closes the loop [194]. Cooperation and commitment from all stakeholders throughout the value chain is necessary to achieve this transition to sustainability, because transparency and traceability are required at all stages. Consumers must know where their clothes come from and which additives were incorporated in the fabric, whether or not the production process was ethical, whether the wastewater was treated properly, and whether the manufacturers used renewable energy and materials. LCAs, social LCAs (SLCAs) and technology assessments can answer these questions by identifying priority areas for intervention and measuring the feasibility of all proposed solutions. In this article, we discuss the outcome of previous LCAs covering polyester garments [8, 29] and textiles in general [13], but these studies do not address the challenge of microfibers [34] and do not address the social aspects of sustainability. It is therefore necessary to prepare inclusive LCAs and SLCAs covering all phases of the polyester value chain, taking into account the different issues described herein, as well as LCAs and SLCAs describing new processes or business models. This comprehensive analysis will provide the guidance needed to ensure meaningful and effective change to improve the sustainability of textiles on a global basis.

## Data Availability

Not applicable.

## Abbreviations

BTEX: Benzene, toluene, ethylbenzene, xylene
GHG: Greenhouse gas
LCA: Life cycle assessment
PET: Polyethylene terephthalate
SLCA: Social life cycle assessment
TPA: Terephthalic acid
WWTP: Wastewater treatment plant
